# UWB electronic positioning error suppression method based on residual weighting

**DOI:** 10.1038/s41598-025-09899-8

**Published:** 2025-07-09

**Authors:** Lin Yang, Bolong Chen, Zhiwei Cao

**Affiliations:** 1 Wuhan Electric Power Technical College, Hubei, 430079 China; 2 College of Intelligent Systems Science and Engineering, Harbin Engineering University, Harbin, 150001 China; 3 Changchuan Technology Co., Ltd., Hangzhou, 150028 China

**Keywords:** Engineering, Electrical and electronic engineering

## Abstract

In the process of construction and operation of electric power facilities, manual inspection is unavoidable, but security incidents happen occasionally. To ensure the safety of workers, ultrawide-band (UWB) positioning technology can be used to locate workers in real time. However, due to the complex environment of electric power facilities, the UWB system does not perform well due to the non-line-of-sight (NLOS) errors. To solve this problem, a UWB positioning algorithm based on Received Signal Strength Indication (RSSI) is proposed to suppress NLOS errors. Determine whether the tag is in an NLOS environment by seeing if the signal strength exceeds the threshold. If the number of remaining nodes is less than 4, the least squares method is used to calculate the position. If the number of remaining nodes is greater than or equal to 4, the NLOS error suppression algorithm based on residual power weighting is used to reduce the positioning error. The high-order weight function is used to assign weights to each LOS node, which makes the weights sensitive to the change of residuals and effectively inhibits errors. Results demonstrate that this algorithm can effectively identify NLOS environments and suppress NLOS error.

## Introduction

With the rapid advancement of modern technology, location-based systems have become a cornerstone for numerous services. In open outdoor environments, Global Navigation Satellite Systems (GNSS) can achieve sub-meter navigation accuracy^1^. However, in complex operational environments such as power facilities, the reliability of GNSS positioning is significantly compromised due to two critical factors. Because of the complex and irregular structural forms of electrical devices, they can block the propagation of electromagnetic wave signals, leading to multipath effects. These operational characteristics collectively degrade signal propagation stability and positioning accuracy in such mission-critical scenarios. Consequently, this limitation has fueled an increasing demand for high-precision indoor positioning technologies^2^.

The widely used Short-range positioning technologies include Radio Frequency Identification (RFID) devices, ZigBee, Bluetooth, Ultra-Wideband (UWB), Wi-Fi, etc^3^. UWB is a wireless communication technique with significant development prospects. As a carrier-less communication technology, UWB uses extremely narrow pulse signals to transmit information. Compared with traditional wireless communication technologies, UWB has many advantages^4^. First, UWB operates at very low power levels, which makes it very energy efficient. Second, it can be resistant to interference, this character ensures reliable communication in complex environments. Finally, UWB signals have high time resolution, which enables remarkably accurate distance measurements and further enhances their versatility and application potential.

With the continuous development of UWB technology, many companies are using UWB technology to introduce low-cost and high-performance solutions. Intel, Apple, and other companies have integrated UWB into their electronic products. Ubisense applies UWB in vehicle positioning scenarios such as parking lots, logistics, etc., and it can reach the metric level of accuracy. Compared with other positioning technologies, UWB stands out due to its superior positioning accuracy and high data transmission efficiency. These advantages make it an ideal choice in positioning applications. In engineering fields, UWB technology has broad applications, including complex electrical construction processes. Through UWB positioning technology, workers in the construction area can be located in real time, making workers safer and enabling smarter management^5^. Moreover, UWB is used for precise positioning of electric power inspection robot. The inspection robot based on UWB positioning can check electric power facilities effectively, thereby significantly reducing the risk of accidental injuries associated with traditional manual inspection methods^6^. UWB can also be used as an electronic fence which provides electronic information support for administration^7^. As a result, UWB positioning technology has become one of the hottest technologies in indoor positioning today^8^.

When using UWB systems for positioning, the primary method is to collect the information from the tags and the base stations and calculate the distances between them using this information. UWB system can calculate the position of the tag through geometric relationships. Normally, the UWB system uses ranging-based positioning methods to determine the location of the tag, which include Time of Arrival (TOA), Angle of Arrival (AOA), Received Signal Strength Indicator (RSSI), and Time Difference of Arrival (TDOA). Therefore, the accuracy of the ranging information will directly affect the positioning accuracy of the entire system^9^. It is essential to ensure that the distance information is accurate^10^. In Line-of-Sight (LOS) conditions, unobstructed signal propagation enables highly accurate ranging measurements. However, when there is an obstacle between the positioning target and the base station, a Non-Line-of-Sight (NLOS) environment is formed. Under NLOS conditions, signal transmission channels are obstructed, and the signal has to be reflected and refracted several times to reach the receiver^11^. Therefore, UWB positioning errors can become substantial in complex NLOS environments.

When all UWB positioning nodes operate under LOS conditions, the least squares method can be used to estimate the target’s position, but when there are nodes that operate under NLOS conditions, using least squares may introduce NLOS errors into the position estimation. In this case, it is necessary to suppress the NLOS error of the UWB technology to improve the positioning accuracy of the UWB in NLOS environment^12^.

Several error suppression algorithms are commonly used to solve this problem, but each has advantages and disadvantages. The first algorithm proposed a localization method based on RSSI fingerprints. Extract the signal strength difference (SSD) fingerprint and RSSI kurtosis feature from the RSSI fingerprint, and construct the Radio Robust image fingerprint (RRIF) through these features. Location estimation is performed using the RRIF and a deep residual network (ResNet). The effectiveness of this algorithm was validated on a public dataset. However, the number of model parameters is relatively large in this method, posing challenges to the computing resources and real-time performance on mobile devices^13^. The second algorithm is based on the information of all nodes, which includes LOS and NLOS nodes. Although the information of NLOS nodes introduces a certain amount of error, this method uses an optimization algorithm to suppress the error and finally calculates the true location of the tag. However, as this method uses an optimization algorithm, it gives the system a high complexity^14^. The third algorithm realizes indoor three-dimensional positioning through multi-classifier fusion named FLMCF. Reduce the positioning error in the vertical direction by conducting classification training between floors. Model training is carried out through multiple classifiers, and the optimal weight set is trained by minimizing the average positioning error. The advantages of each classifier are fully combined to improve the positioning accuracy. Finally, the optimal weights are selected through the Reliability Fusion Weight Selection (RFWS) algorithm, the outputs of multiple classifiers are fused, and the positions are estimated accordingly. However, the selection of weight constraint parameters relies on experience, which may cause subjective deviations^15^. The fourth algorithm is based on a weighting method, which first analyses the error of each node and then assigns an appropriate weight to the information of each node according to the magnitude of the error. This algorithm makes use of the information of LOS and NLOS nodes, giving higher weight values to LOS nodes and lower weight values to NLOS nodes, making full use of the information of LOS nodes, and can effectively improve positioning accuracy^16^. However, the key to this weighting-based method lies in the selection of weights, which can be allocated scientifically and reasonably in order to make full use of the ranging information of each node, and the positioning effect of the system will be better. The fifth algorithm builds upon the robust Kalman filter (RKF). This algorithm proposes an improved RKF algorithm (RKF-SW) to enhance the accuracy and reliability of UWB positioning, particularly in complex scenarios with obstructions. The study builds on the traditional RKF and introduces sample quantile and sliding window methods to further optimize outlier detection and handling. The improved RKF algorithm has a strong ability to identify outliers. Therefore, it can enhance the positioning accuracy of UWB systems in NLOS environments^17^. However, the impact of the network geometry of the base station is not sufficiently explored. Different base station geometries may influence the ranging and positioning results. The sixth algorithm presents a novel approach for UWB NLOS/LOS classification by using deep learning techniques, specifically combining Convolutional Neural Networks (CNN) with Long Short-Term Memory (LSTM)^18^. In this paper, the authors use UWB channel impulse response (CIR) data as input to the CNN directly, and the CNN can automatically extract features. These features will be fed into an LSTM for the classification of LOS and NLOS signals. The approach was tested using datasets from seven different indoor locations, which were collected from open-source datasets. The CNN-LSTM model demonstrates superior performance compared to conventional machine learning methods such as Support Vector Machine (SVM) and Multi-Layer Perception (MLP). This algorithm requires a high-quality dataset for training and must deal with potential overfitting issues carefully, particularly in specific environments. Additionally, the model must have strong generalization ability to effectively adapt to complex indoor environments. The seventh algorithm introduces new error bounds for TOA-based positioning in NLOS conditions, which enable better estimation and reduction of these errors^19^. Moreover, these new error bounds are integrated with positioning algorithms to improve the robustness and accuracy of positional estimation in challenging environments. However, the integration of error bounds and positioning algorithms may increase the computational complexity, potentially limiting their application in real-time systems. Therefore, this paper proposes an RSSI-based NLOS error suppression algorithm to suppress NLOS errors and improve the positioning accuracy of UWB in the NLOS environment. The major innovations of this paper lie in: (1) utilizing RSSI thresholds to determine whether base stations are in NLOS environments; (2) adaptively selecting appropriate algorithms based on the number of effective base stations; and (3) assigning weights to each combination using the residuals of temporary position estimates obtained through base station localization.

## Algorithm for suppressing NLOS errors

In practical positioning applications, two main types of errors can cause a reduction of UWB positioning accuracy: The first type results from inaccuracies in distance and angle measurements made by the positioning terminals or base stations, which are primarily hardware limitations. These errors generally have little impact, and mathematically, these errors follow a zero-mean Gaussian distribution. So, it’s not a major factor. The second type of error is caused by obstacles that block the transmission of UWB signals. These errors cause significant distance measurement deviations. That is the NLOS error. These NLOS errors substantially affect the performance of UWB positioning systems. Unfortunately, many obstacles can block UWB signals in actual scenarios. In engineering practice, to ensure the positioning accuracy of UWB systems, it is usually necessary to minimize the effect of NLOS errors.

Since the position information in UWB systems is based on the ranging data from base stations, the veracity of this ranging information is critical to the positioning accuracy of the UWB system. In LOS environments, UWB signal propagation is unimpeded, and the obtained ranging data is generally more accurate. However, when obstacles exist between the positioning target and the base station, an NLOS environment is formed. In this case, signals experience refraction and diffraction during propagation, resulting in delays in arrival time. This delay causes the error of measurement, which makes the measurement bigger than actual distance and the UWB positioning accuracy will be affected by this error, even causing the location failure.

When all UWB positioning nodes are in the LOS environment, the tag’s position can be accurately estimated by using the least squares method. However, if some nodes are in an NLOS environment, the least squares method will introduce NLOS errors into the position estimation. To solve this problem, it is necessary to suppress NLOS errors to improve the positioning accuracy in NLOS environments.

The residual in mathematical statistics is the difference between the actual observed value and the estimated value, which reflects the error caused by the prediction using the estimated regression equation. For two-dimensional UWB positioning, a minimum of three base stations’ range measurements are required^20^. Firstly, every three of the base stations are combined, and their position estimates are found for each combination by least squares. The residuals for each group are calculated by averaging the position estimates of all combinations as provisional observations.

When multiple base stations are used for positioning, if every three base stations are grouped, then the group containing the NLOS data introduces NLOS errors into the position estimation, and there are large errors in the calculation of the provisional observations. Since the signal strength of UWB attenuates with effects such as distance and reflections, it maintains a consistent trend across diverse environments and is less affected by external conditions. Additionally, the signal strength is technically easy to identify. Therefore, this paper employs an RSSI-based method for NLOS error mitigation^21^. The UWB base station used in the experiment can measure the strength of the signal emitted by the UWB tag when it reaches the base station. The signal strength information measured by the base station mainly includes the first path signal strength and the total received signal strength. The first signal strength received by the base station after the UWB tag has emitted a signal is the first path signal strength, generally denoted by RSSIFP. When the signal reaches the base station after refraction and reflection, the signal strength received by the base station is called the total received signal strength, which is generally expressed as RSSIRX^22^. Based on this, the difference between the first path signal strength and the total received signal strength is used as the judgment condition for LOS/NLOS, as shown in Eq. (1).

1 $$RSS{I_{\text{R}\text{X}}} - RSS{I_{\text{F}\text{P}}}=\left\{ {\begin{array}{*{20}{c}} { \leqslant Threshold,LOS} \\ {>Threshold,NLOS} \end{array}} \right.$$

**Fig. 1:**
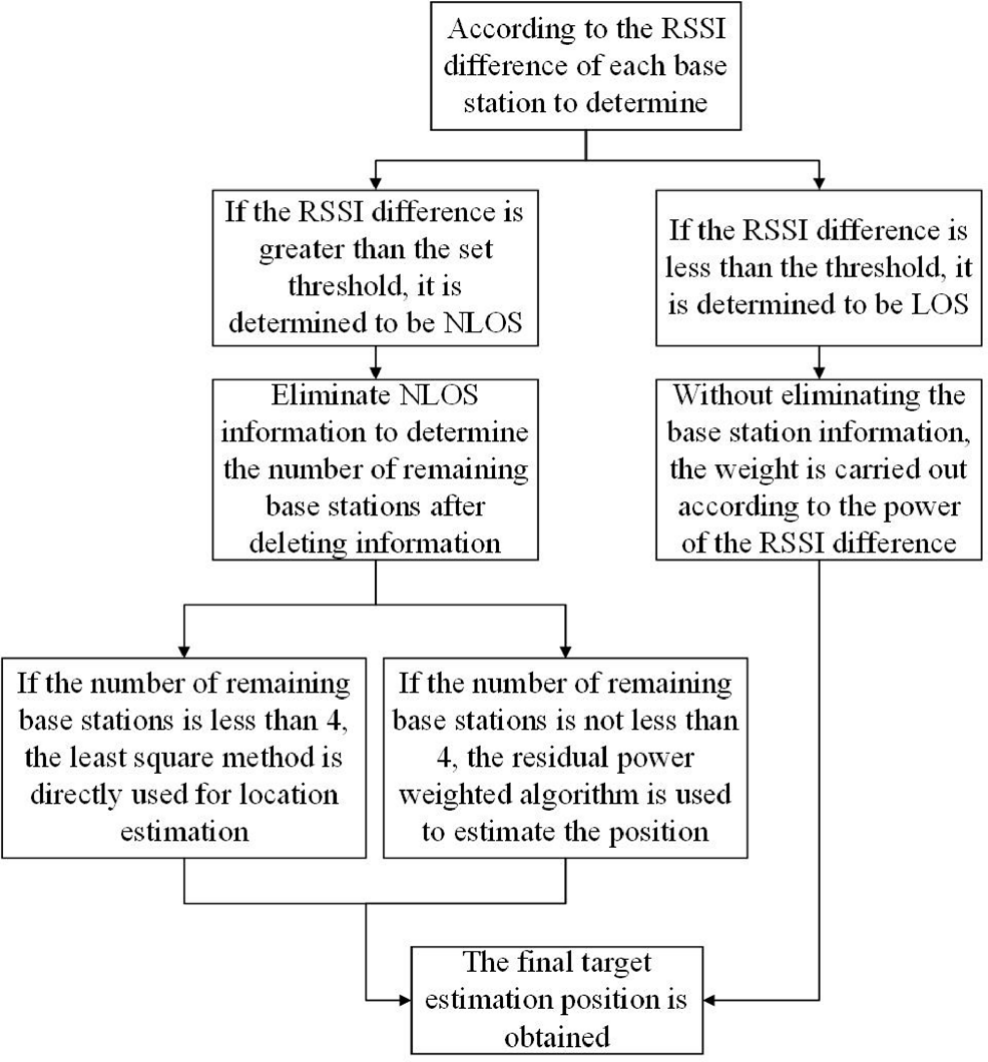
Flow chart of NLOS error suppression algorithm.

Where Threshold is the set threshold of RSSI difference. When the RSSI difference does not exceed the set threshold, indicating minimal NLOS error, there is no need to reject the range value. The power of the RSSI difference is used as the weight of each base station and the range value of all base stations is used for positioning. However, when the RSSI difference exceeds the threshold, signifying significant NLOS error that cannot be adequately suppressed through weighting alone, the corresponding base station is identified as operating in NLOS conditions and its range values are excluded from the positioning calculation. In such cases, positioning proceeds using only the remaining LOS base stations’ range values. If the number of remaining base stations is less than 4 after NLOS exclusion, then the least squares method is used to estimate the position. If four or more valid stations are available, a NLOS error suppression algorithm based on power-weighting of the residuals is used to reduce the positioning error^23^. Figure 1 shows the flow chart of the NLOS error suppression algorithm.

Assuming that the range values from N base stations are used for positioning after excluding the NLOS data, then the provisional observations are:

2 $${X_0}=\frac{{\sum\nolimits_{{i=1}}^{M} {{X_i}} }}{M}$$

Where, $M=C_{N}^{3}(N \geqslant 4)$ is the number of combinations, and X_i_ is the position estimation result of the i th combination. By analyzing each combination, we can deduce that the smaller the residual value of the provisional position, the smaller the estimated position error for that particular combination. On this basis, it is possible to estimate the provisional position for each combination and use the resulting residuals of the provisional position as an important basis for assigning weights to each combination. This strategy can reduce the adverse effects of the NLOS environment on the positioning system and improve positioning accuracy. After assigning weights to each group based on the residual values, the position coordinates of the tags are finally calculated as shown in (3):

3 $$\hat {X}=\frac{{\sum\nolimits_{{i=1}}^{M} {{W_i}(\Delta ){X_i}} }}{{\sum\nolimits_{{i=1}}^{M} {{W_i}(\Delta )} }}$$

**Fig. 2:**
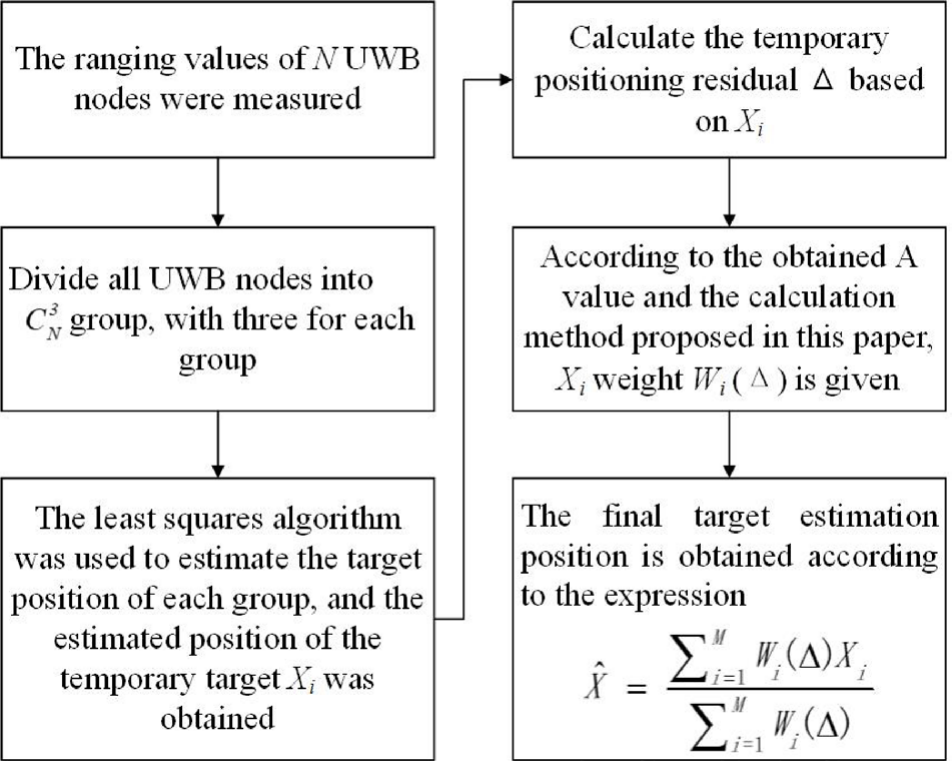
Flow chart of residual power weighting algorithm.

Where W_i_(△) represents the weighting function associated with the temporary positioning residual value, and Xi represents the temporary position estimate for the *i* th group. Figure 2 shows a flow chart of the residual power weighting algorithm.

In the traditional weighting algorithm, the weights are distributed directly using the reciprocal of the residuals. However, this approach exhibits significant limitations in environments with substantial NLOS errors. The fundamental issue lies in the algorithm’^24^. To overcome this situation, this paper proposes a scheme to use higher-order power functions as weighted functions, and the function values of these functions change more dramatically.

4 $${W_i}(\varDelta )={(1/\varDelta )^n},n \geqslant 2$$

**Fig. 3:**
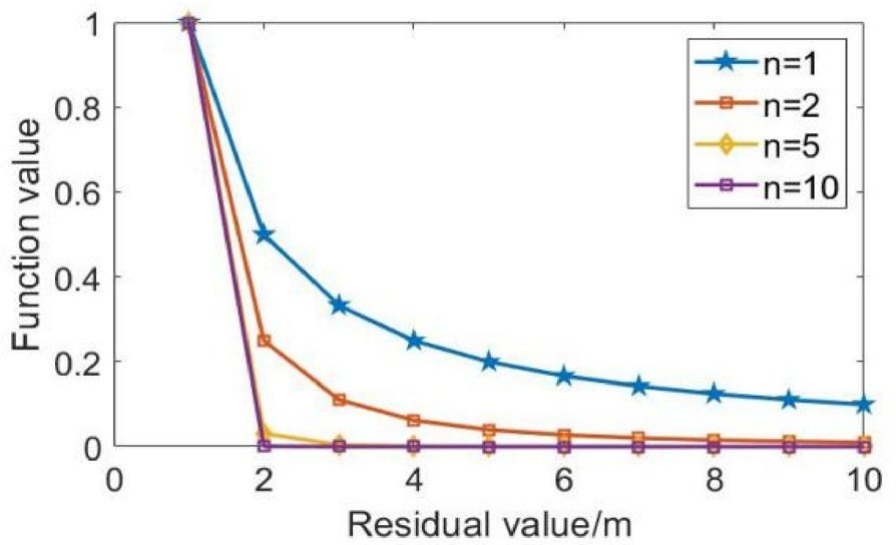
Numerical changes of different weight functions.

The residual value (denoted by△) represents the position estimation error. Analysis of the weighting function curves reveals that higher exponents (n) produce more pronounced curve variations at small residual values, indicating enhanced sensitivity to residual changes. This relationship demonstrates that when NLOS errors are large (corresponding to large residuals), the weighting function yields smaller, more gradual values, thereby significantly improving the system’s NLOS error suppression capability. Furthermore, Fig. 3 analysis shows that the curves to the power of 5 and 10 tend to coincide, and this paper selects for positioning.

Using the RSSI-based NLOS error suppression algorithm, the NLOS data is first eliminated by comparing the difference of RSSI of each base station, and for the case of four base stations, the ranging value of one base station is eliminated. The remaining three base stations are used for positioning, and the least squares method is used for position estimation. For the case of five base stations, if the ranging values of the two base stations are eliminated, the remaining three base stations are used for positioning, and the least squares method is also used for position estimation. If one base station is eliminated, the residual power weighting algorithm can be used to reduce the positioning error of the UWB system in the NLOS environment and improve the positioning accuracy by using the ranging values of the remaining four base stations.

## UWB positioning experiment and analysis

**Fig. 4:**
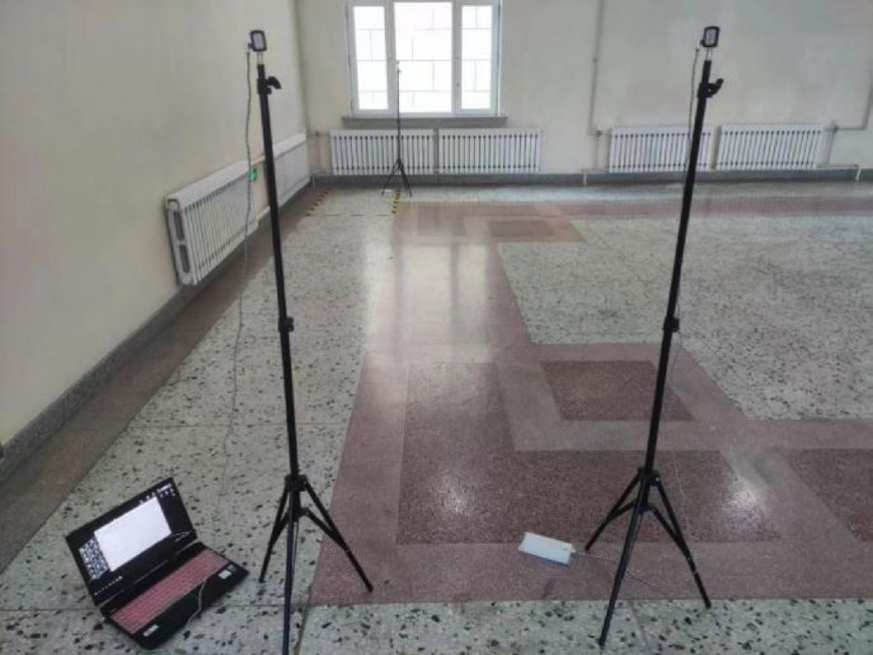
Experimental environment.

The LinkTrack UWB positioning system developed by Nooploop was used for the experiment. In the line-of-sight environment, the positioning system can reach 0.1 m in the indoor environment with two-dimensional positioning accuracy, positioning frequency up to 200 Hz, the tag real-time output to each base station ranging value and its position coordinates and other information, the use of the base station can collect all the positioning tags in real-time to reach the ranging value of each base station and the coordinate information of the tag. The experimental settings are depicted in Fig. 4.

### Dynamic positioning experiments in the LOS environment

The base stations were arranged within the experimental site and their positions were adjusted accordingly. Three base stations formed a near-equilateral triangle; four base stations were positioned in a square arrangement; and five base stations were deployed in a regular pentagon. To minimize the influence of base station arrangement on positioning results, all base stations were adjusted to the same height level. During testing, a mobile label traversed a predefined trajectory while continuously collecting ranging data from all base stations. Eight characteristic points were selected along the trajectory to analyze the positioning system’s performance.

**Fig. 5:**
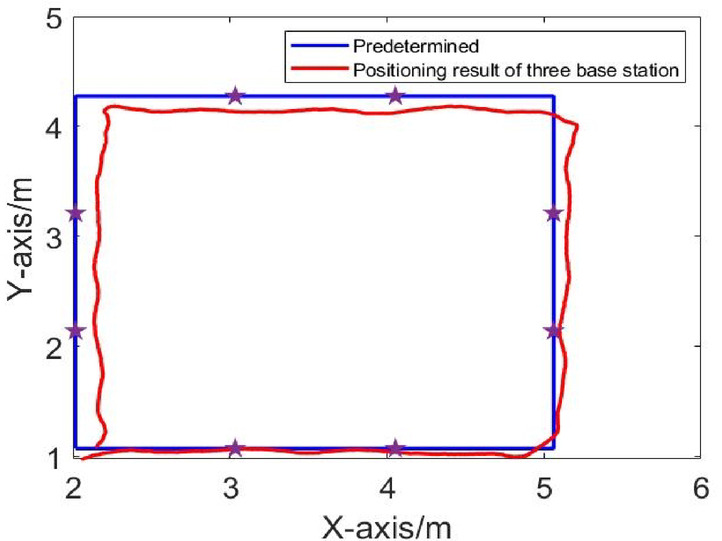
Dynamic positioning trajectory in LOS environment of three base stations.

**Fig. 6:**
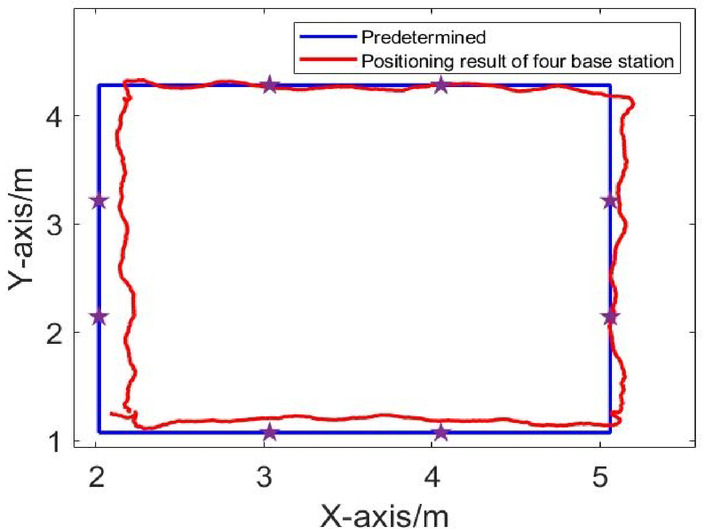
Dynamic trajectory in LOS environment of four base positioning stations.

**Fig. 7:**
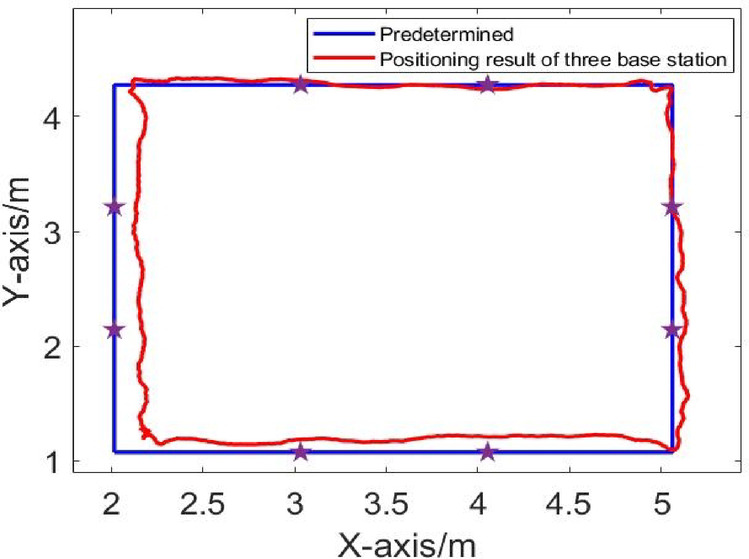
Dynamic trajectory in LOS environment of four base positioning stations.

**Fig. 8:**
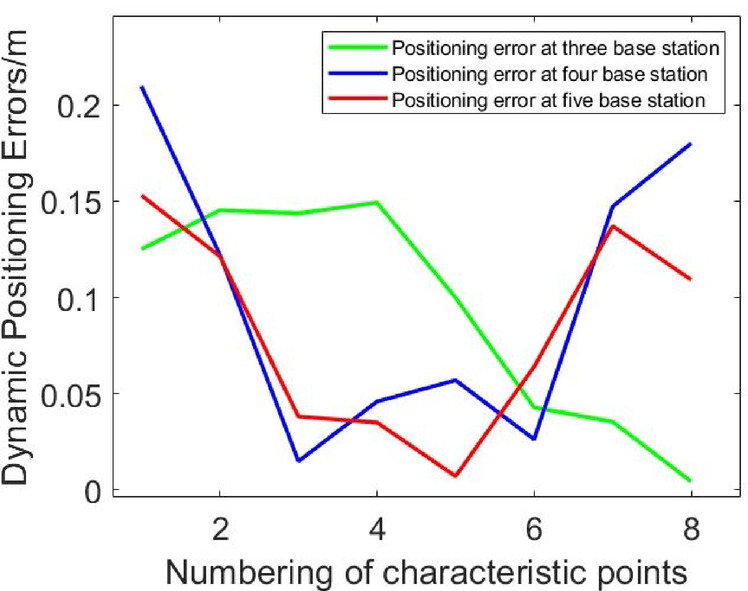
Comparison of dynamic positioning errors at three, four, and five base stations.

The positioning algorithm is used to solve the driving trajectory, and the positional error between the actual trajectory and the intended trajectory is calculated. The positioning results in LOS environment are shown in Figs. 5, 6, 7. Comparison of Dynamic Positioning Errors at three, four, and five base stations are shown in Fig. 8. According to Table 1, in the LOS environment, the maximum position error of dynamic positioning of the three-base station is 0.1492 m, and the minimum position error is 0.0045 m; the maximum position error of dynamic positioning of the four-base station is 0.175 m, and the minimum position error is 0.0036 m; the maximum position error of dynamic positioning of the five-base station is 0.153 m, and the minimum position error is 0.0072 m. Although the maximum positional error of the three-base station setup is smaller than that of the four-base station setup and five-base station setup, the overall RMS error presents a different perspective. The RMS error of the three-base station setup is 0.1077 m, and the RMS error of the four-base station setup and five-base station setup are 0.0996 m and 0.0972 m, respectively. This indicates that the dynamic positioning accuracy increases with the increase of the number of base stations in LOS conditions, and the five-base station setup achieves the highest accuracy.

**Table 1:** Comparison of dynamic positioning errors at three, four, and five base stations.

Type of error	Three base stations	Four base stations	Five base stations
Minimum positional error (m)	0.0045	0.0036	0.0072
Maximum positional error (m)	0.1492	0.175	0.153
Root mean square error (m)	0.1077	0.0096	0.0972

### Dynamic positioning experiments for four- and five-base stations in NLOS environment

Four UWB base stations are erected clockwise in a rectangular area with a width of 7 m from east to west and a length of 7 m from north to south, and the four base stations are fixed on a three-legged bracket with a height of 1.6 m, of which the No. 1 base station is the main base station, and the No. 2, 3 and 4 base stations are secondary base stations, the secondary base station is powered by mobile power, and the main base station is directly connected to the computer serial port. The frequency of the UWB acquisition data is set to 50 Hz. The connection between the main base station 1 and the secondary base station 4 is the X axis, and the Cartesian coordinate system is established between the main base station 1 and the secondary base station 2 for the Y axis. The coordinates of each sub-base station relative to the main base station are measured with a meter measure, and the corresponding coordinates are written to the parameter settings of the main base station. The coordinates of each base station are: No. 1 base station (0,0), No. 2 base station (0,7), No. 3 base station (7,7) and No. 4 base station (7,0). In the scenario of five base stations, the coordinates of each base station are: NO. 1 base station (2,0), NO. 2 base station (0,3.5), NO. 3 base station (4,6), NO. 4 base station (8,3.5) and NO. 5 base station (6,0). During the experiment, a base station was artificially occluded to achieve a non-line-of-sight effect.

**Fig. 9:**
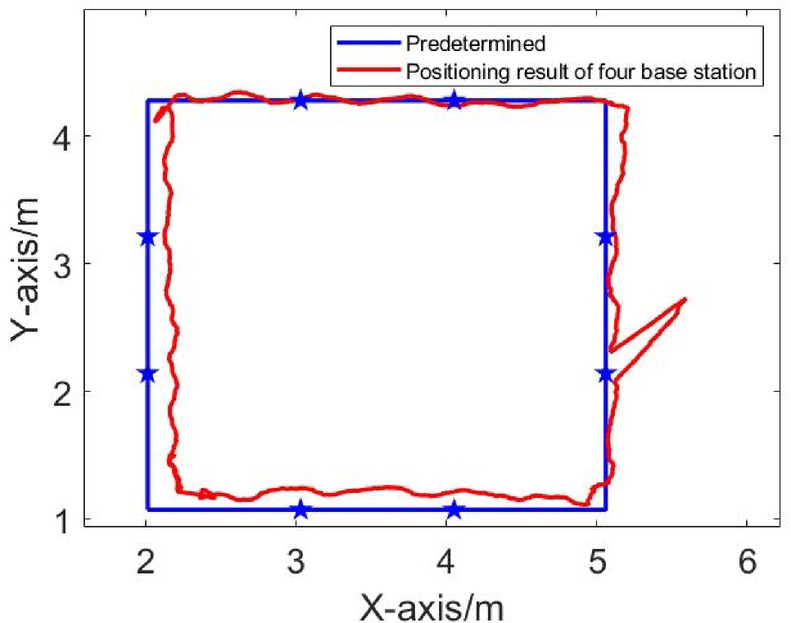
Dynamic trajectory in NLOS environment of four base positioning stations.

**Fig. 10:**
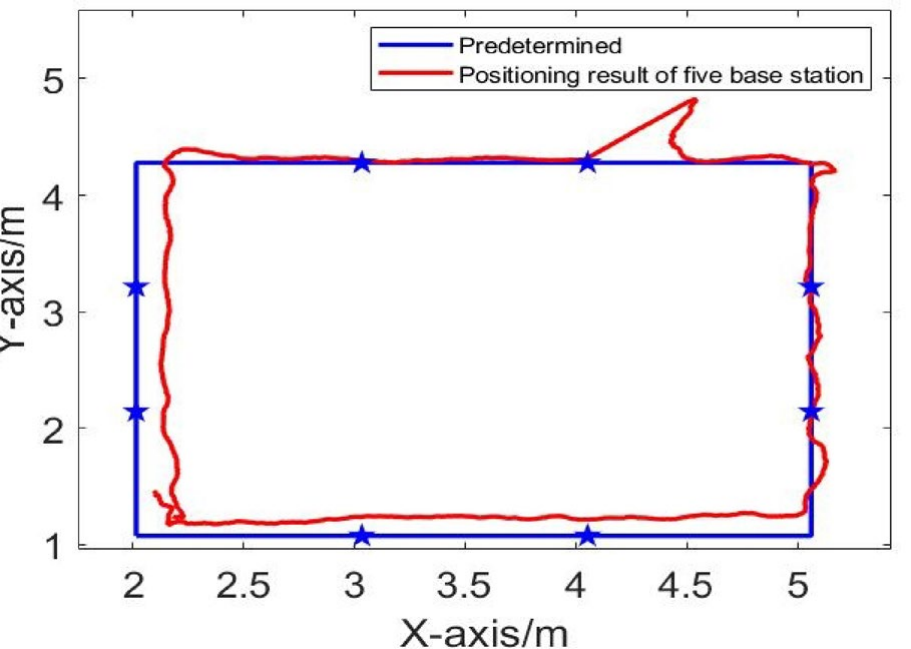
Dynamic trajectory in NLOS environment of five base positioning stations.

From Figs. 9 and 10, it can be seen that when there is no obstruction to the UWB base station, the UWB positioning trajectory can basically reproduce the actual driving trajectory, demonstrating high positioning accuracy. However, when the UWB base station is obscured by obstacles, the trajectories deviate significantly.

**Fig. 11:**
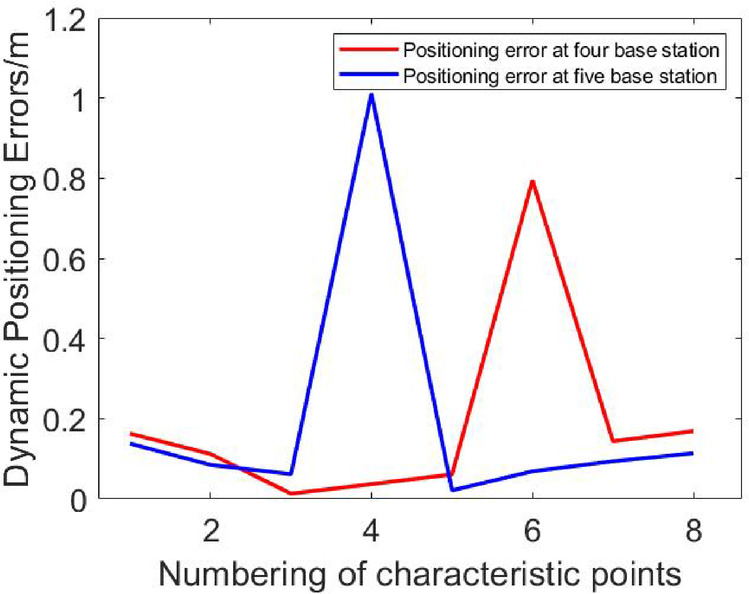
Comparison of dynamic positioning errors at four and five base stations.

According to Fig. 11, in the NLOS environment, the maximum positioning error of the four-base station setup is 0.7949 m, and the maximum positioning error of the five-base station setup is 1.0114 m. Due to obstacles, both four-base station and five-base station show a large positioning error in UWB positioning. It can be seen that increasing the number of base stations cannot solve the problem of reduced positioning accuracy of the UWB system caused by obstructions. To address this, an NLOS error suppression algorithm is applied. This algorithm eliminates NLOS data by comparing the RSSI differences across base stations.

**Fig. 12:**
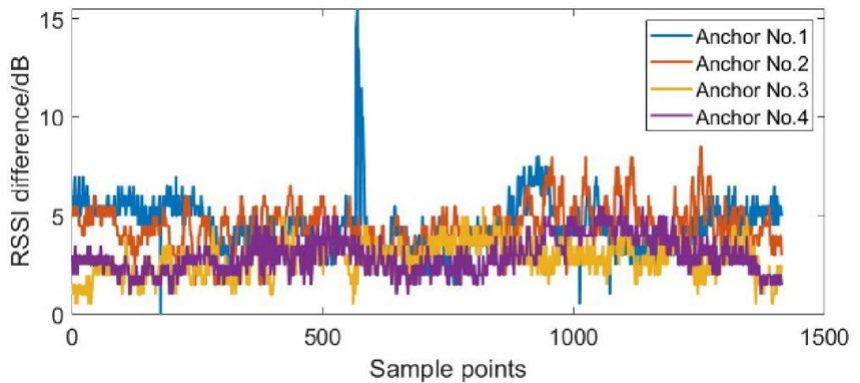
The RSSI difference in the dynamic positioning experiments of four base stations.

**Fig. 13:**
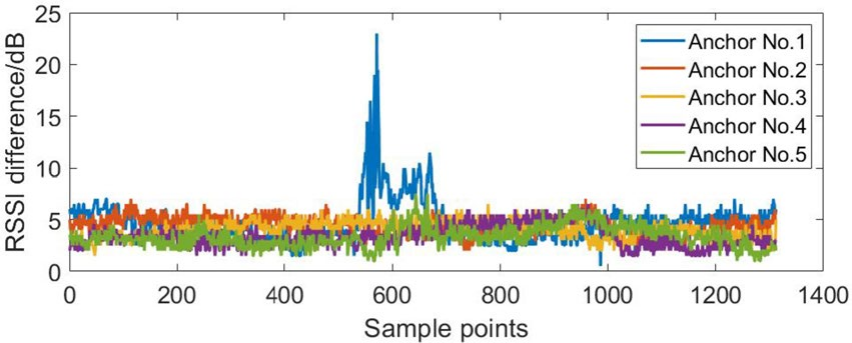
The RSSI difference in the dynamic positioning experiments of five base stations (block No.1 base station).

**Fig. 14:**
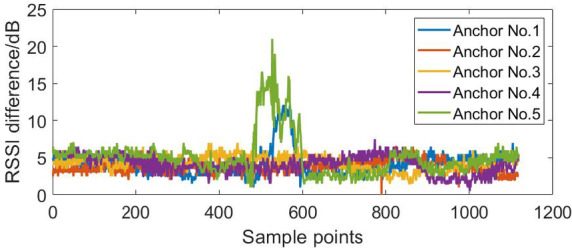
The RSSI difference in the dynamic positioning experiments of five base stations (block No.1 and No.5 base stations).

The NLOS error suppression algorithm based on RSSI is applied to the dynamic positioning of four base stations and five base stations in the NLOS environment. According to the RSSI differences in the dynamic positioning experiments shown in Figs. 12, 13 and 14, the Threshold was selected as 8.

**Fig. 15:**
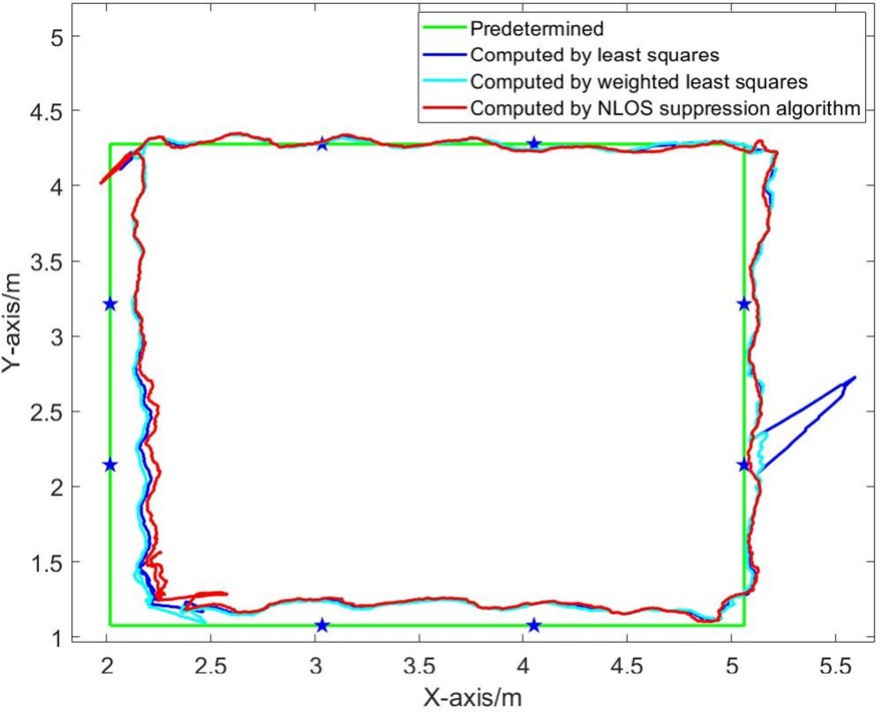
Dynamic positioning trajectory in NLOS environment of four base stations.

**Fig. 16:**
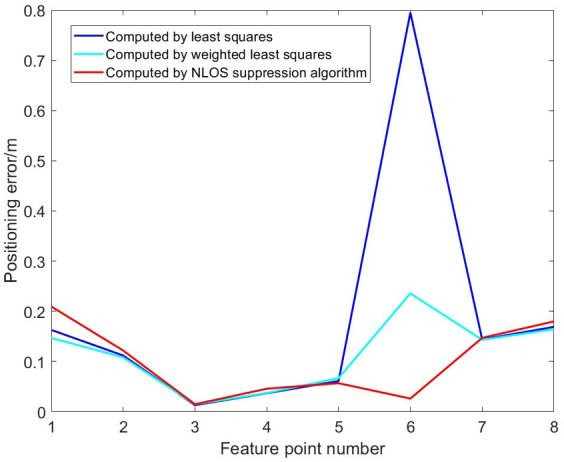
Comparison of position errors in NLOS environment of four base stations.

**Fig. 17:**
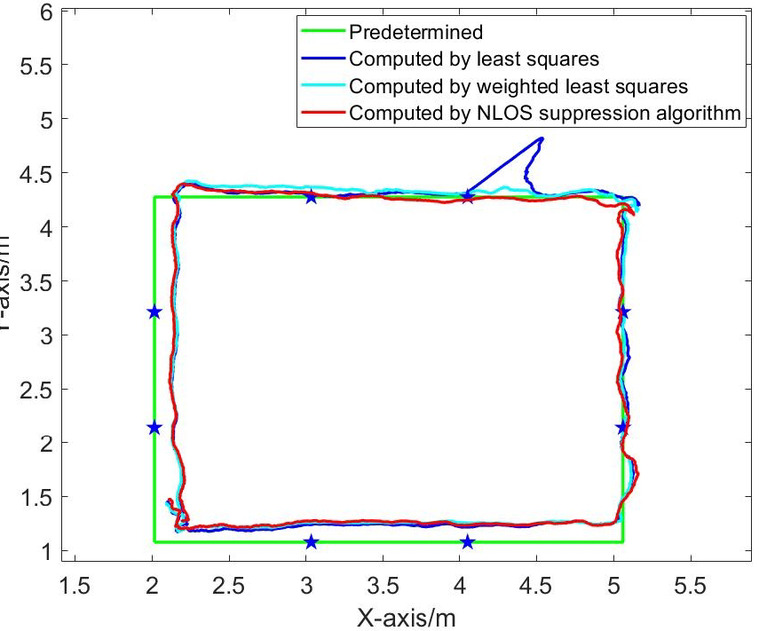
Dynamic positioning trajectory in NLOS environment of five base stations (blocking No. 1 base station).

**Fig. 18:**
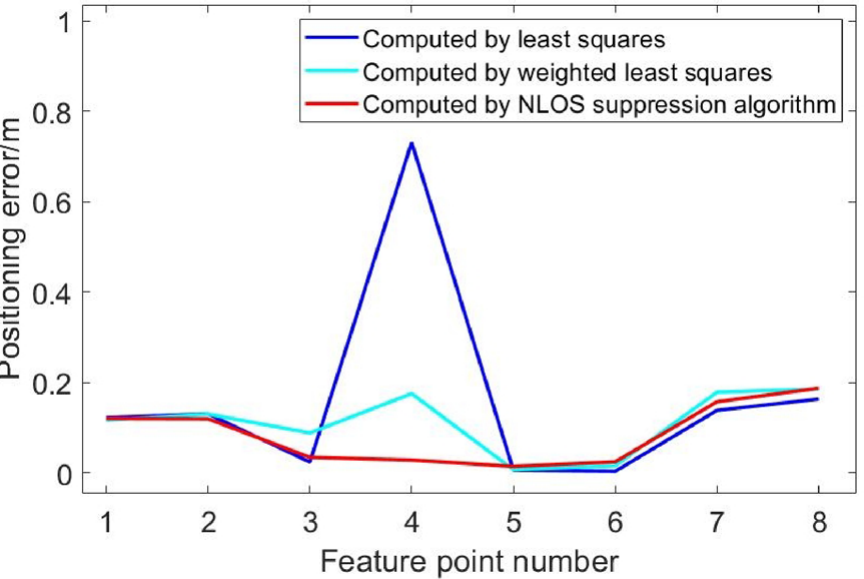
Comparison of position errors in NLOS environment of five base stations (blocking No. 1 base station).

**Fig. 19:**
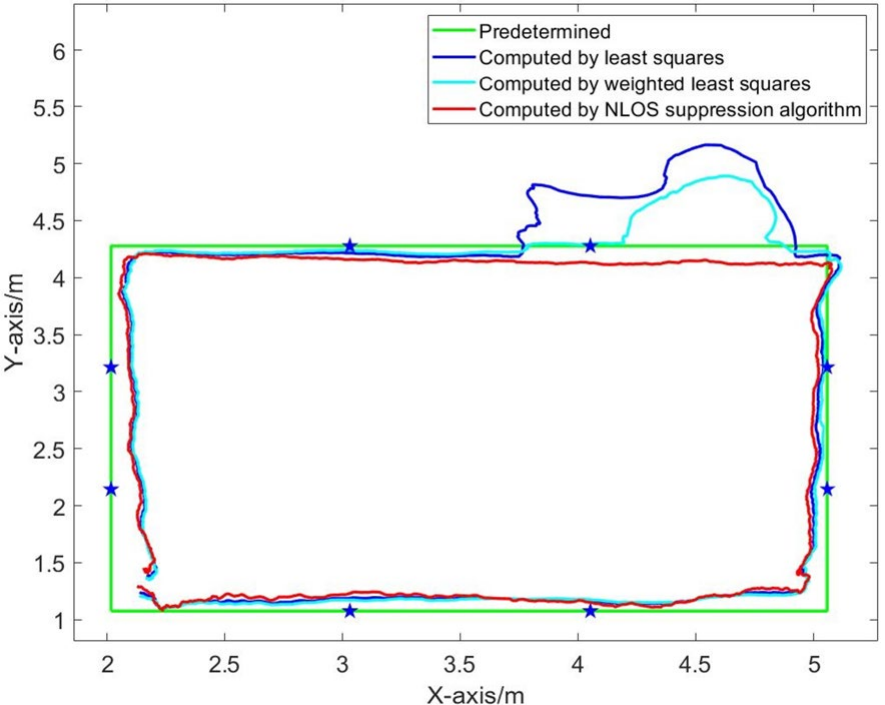
Dynamic positioning trajectory in NLOS environment of five base stations (blocking No. 1 and No. 5 base stations).

**Fig. 20:**
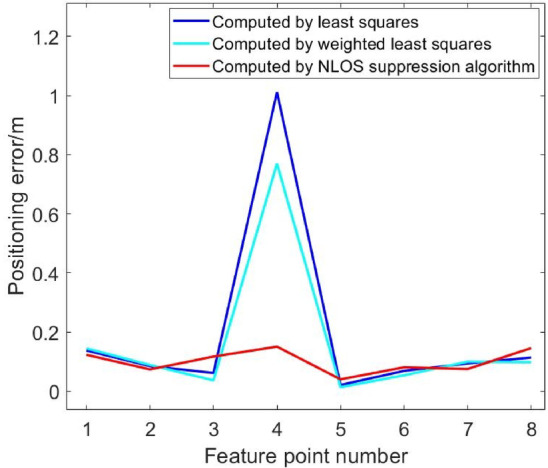
Comparison of position errors in NLOS environment of five base stations (blocking No. 1 and No. 5 base stations).

Figures 15, 16, 17, 18, 19 and 20 show the solution results of the commonly used least square method, weighted least square method and NLOS error suppression algorithm. It can be seen that when using the least square method for calculation, the maximum position errors are 0.7949 m, 0.7307 m and 1.011 m respectively, and the influence of NLOS error cannot be avoided. When using the weighted least squares method for calculation, the maximum position errors are 0.2358 m, 0.1761 m and 0.7698 m respectively. The weighted least squares method can reduce the influence of NLOS error to a certain extent, but it will still lower the positioning accuracy. After adopting the NLOS error suppression algorithm, the maximum position error under the four base stations is 0.2096 m, and the maximum position error under the five base stations is 0.188 m and 0.151 m, respectively, and the position error caused by obstacle occlusion is well suppressed, and the UWB positioning solution trajectory that deviates too much from the predetermined trajectory is corrected back to the predetermined trajectory, which effectively reduces the influence of NLOS data on the positioning accuracy. In NLOS environments, the positioning performance with five base stations is better than that with four base stations. When five base stations are used, even if two base stations are in NLOS environment, this system still has a better positioning accuracy by removing these NLOS data. This experimental result demonstrates that the RSSI-based NLOS error suppression algorithm proposed in this paper effectively reduces the impact of NLOS data on positioning accuracy.

## Conclusion

In this paper, an NLOS error suppression algorithm based on RSSI is proposed, which adopts a power-weighting algorithm based on RSSI difference for the measured values that are less affected by NLOS error, eliminates the measured values that are greatly affected by NLOS error, and uses other measured values to use the residual power weighting algorithm to treat the weighted estimation of the location node position. Experimental results show that the RSSI-based NLOS error suppression method proposed in this paper can effectively eliminate the wild value in the positioning process, significantly reduce the positioning error of UWB in the NLOS environment, and improve the positioning accuracy and reliability of the system.

## Data Availability

The data involved in this article are generated by real experiments and collected from experiments. If necessary, they can be acquired from the corresponding author.
